# HSP Transcript and Protein Accumulation in Brassinosteroid Barley Mutants Acclimated to Low and High Temperatures

**DOI:** 10.3390/ijms21051889

**Published:** 2020-03-10

**Authors:** Iwona Sadura, Marta Libik-Konieczny, Barbara Jurczyk, Damian Gruszka, Anna Janeczko

**Affiliations:** 1 Polish Academy of Sciences, The Franciszek Górski Institute of Plant Physiology, Niezapominajek 21, 30-239 Kraków, Poland; i.sadura@ifr-pan.edu.pl (I.S.); m.libik@ifr-pan.edu.pl (M.L.-K.); 2 Department of Plant Physiology, University of Agriculture in Kraków, Podłużna 3, 30-239 Kraków, Poland; barbara.jurczyk@urk.edu.pl; 3 Institute of Biology, Biotechnology and Environmental Protection, Faculty of Natural Sciences, University of Silesia, Jagiellońska 28, 40-032 Katowice, Poland; damian.m.gruszka@gmail.com

**Keywords:** brassinosteroids, acclimation process, small HSPs (sHSPs), HSP70, HSP90, temperature stress

## Abstract

In temperature stress, the main role of heat-shock proteins (HSP) is to act as molecular chaperones for other cellular proteins. However, knowledge about the hormonal regulation of the production of the HSP is quite limited. Specifically, little is known about the role of the plant steroid hormones—brassinosteroids (BR)—in regulating the HSP expression. The aim of our study was to answer the question of how a BR deficit or disturbances in its signaling affect the accumulation of the HSP90, HSP70, HSP18, and HSP17 transcripts and protein in barley growing at 20 °C (control) and during the acclimation of plants at 5 °C and 27 °C. In barley, the temperature of plant growth modified the expression of HSPs. Furthermore, the BR-deficient mutants (mutations in the *HvDWARF* or *HvCPD* genes) and BR-signaling mutants (mutation in the *HvBRI1* gene) were characterized by altered levels of the transcripts and proteins of the HSP group compared to the wild type. The BR-signaling mutant was characterized by a decreased level of the *HSP* transcripts and heat-shock proteins. In the BR-deficient mutants, there were temperature-dependent cases when the decreased accumulation of the *HSP70* and *HSP90* transcripts was connected to an increased accumulation of these HSP. The significance of changes in the accumulation of HSPs during acclimation at 27 °C and 5 °C is discussed in the context of the altered tolerance to more extreme temperatures of the studied mutants (i.e., heat stress and frost, respectively).

## 1. Introduction

While abiotic stresses, including extreme temperatures, are a natural part of changing environmental conditions, they are also a serious problem for agriculture. Low- and high-temperature stresses are considered to be some of the major sources of abiotic stress for crop plants [1], and they cause yield losses, among others, in cereals [2]. Plants can acclimate to extreme temperatures (such as frost or heat) via temporary exposure to stress factors of a lower intensity such as cold or warm conditions [3,4]. Cold acclimation is particularly important for winter plants. Temperature stress initiates metabolic changes in plants and activates defense mechanisms such as an enhanced expression of the stress-responsive genes including the genes coding proteins from the protective group, which are called heat-shock proteins (HSPs) [5,6,7]. These proteins were first discovered in heat-treated plants and five classes of HSP are now known. They are characterized by their different molecular weights, namely, HSP100, HSP90, HSP70, HSP60, and small heat-shock proteins (sHSP) [5,6,7]. The main role of HSPs is to act as molecular chaperones. They are responsible for regulating protein folding, as well as its accumulation, location, translocation, and degradation in all plant and animal species [6,7,8]. In addition to being molecular chaperones, HSPs also have other functions. HSP90 plays an important role in the functioning and translocation of signaling proteins (e.g., kinases, calmodulin, actin) (for a review, see Al-Whaibi [6]; Park and Seo [7]), while HSP70 is thought to be the most abundant heat-shock protein in eukaryotic cells. It is responsible for protecting plant cells from the damaging effects of heat stress, and it is also thought that it assists denatured protein refolding and prevents its aggregation [9]. The main functions of sHSPs are to degrade any misfolded proteins and to prevent irreversible unfolding or incorrect protein aggregation. They are also thought to play a role in acclimating plants to high-temperature stress. Unlike other HSPs, the activity of sHSPs is independent of ATP (for a review, see Al-Whaibi [6]; Park and Seo [7]). Horváth et al. [10] showed that sHSPs bind to specific domains that are located in cell membranes and modulate their properties and physical states. The cell membrane is an important element in counteracting the negative effects of abiotic stresses (including extreme temperatures) and, according to the work of Horváth et al. [10], cell membranes are considered to be “thermal sensors” (membrane sensor hypothesis); thus, they are thought to be the main cause of many of the metabolic changes within a cell, including the expression of certain genes. Like physiological/biochemical processes, the expression of HSPs may be regulated/modulated by phytohormones. However, knowledge about the hormonal regulation of the production of the HSP production is quite limited. Little is known about the role of the plant steroid hormones, brassinosteroids (BRs), in the expression of HSPs. BRs were discovered in *Brassica napus* pollen in the 1970s [11,12]. BRs occur in almost all parts of a plant and, to date, about 70 BRs were isolated. BRs can prevent or reduce injuries caused by many environmental stresses including extreme temperatures [13,14]. In plants that are exposed to high/low temperatures, the application of BR changes the physicochemical properties of the plant plasma membrane, regulates photosynthesis and sugar production, and generally controls the various directions of the metabolism of plants through its interactions with other phytohormones (for a review, see Sadura and Janeczko [14]). Studies on BR mutants revealed that the endogenous content of BRs or disturbances in BR signaling have an impact on the temperature stress tolerance of *Arabidopsis thaliana*, tomato, and barley plants [15,16,17]. BR mutants with a disturbed BR signaling or with a BR deficit resulting from mutations of the gene encoding enzymes that control the specific steps of BR biosynthetic pathways are generally good models for studying the role of these phytohormones in plants. Therefore, the plant material that was used in the present study included the barley BR mutants 522DK, BW084, and BW312 [18,19]. The 522DK mutant, which was derived from a Delisa cultivar, was induced via chemical mutagenesis in the Delisa cultivar; it has a mutation in the *HvDWARF* gene encoding brassinosteroid C6-oxidase, which is involved in BR biosynthesis [18,20]. This mutation is connected to a lower content of castasterone, brassinolide, and 28-homocastasterone by about 60%, 85%, and 25%, respectively, in the 522DK mutant compared to the wild-type Delisa [17]. The BW084 and BW312 mutants are semi-dwarf near-isogenic lines (NILs) that were derived from a Bowman cultivar [19]. The BW084 (*brh13.p*) carries a mutation in the *HvCPD* gene, which encodes the barley C-23α-hydroxylase cytochrome P45090A1 (CYP90A1) that catalyzes the early stages of BR biosynthesis. This mutation causes a significantly lower level of castasterone and brassinolide (below the detection limit) and an 18% lower content of 28-homocastasterone than the wild-type Bowman cultivar [17]. The BW312 (ert-ii.79) mutant has a double substitution (CC1760/1761AA) in the *HvBRI1* gene encoding the BR receptor kinase, BRI1, which results in a disturbance in the binding of the BR molecules, as well as a significantly higher content of BR [17,19]. After acclimation at 27 °C, all these mutants were characterized by a higher tolerance to high temperatures (38–45 °C) [17]. However, after acclimation at 5 °C, the frost tolerance of mutants was unchanged (522DK, −8 °C), slightly higher (522DK, −6 °C) or lower (BW084 and BW312, −6 °C and −8 °C) [17]. Studies of the expression of heat-shock proteins could, thus, provide knowledge about the hormonal regulation of the production of HSPs (the role of BRs) and could potentially help explain the molecular background of the changed tolerances to temperature stress of BR mutants. The aim of the study was to answer the following main question: How do a BR deficit and disturbances in its signaling affect the accumulation of the HSP90, HSP70, HSP18, and HSP17 transcripts and protein in barley that is grown at 20 °C (control) and during the acclimation of plants at 5 °C and 27 °C?

## 2. Results and Discussion

### 2.1. Presence of Heat-Shock Proteins in Barley Membrane and Cytosolic Fractions

**Figure 1:**
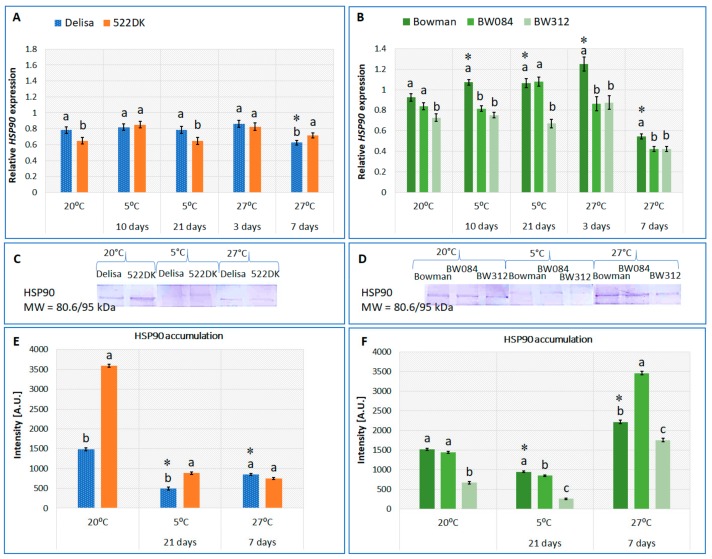
Changes in relative transcript level of heat-shock protein 90 (*HSP90*) (**A**,**B**) and the accumulation of HSP90 (**C**–**F**) in the barley cell membrane fraction isolated from the cultivar (cv.) Delisa, 522DK mutant, cv. Bowman, and the BW084 and BW312 mutants growing in different temperature conditions. The transcript levels are presented as the fold change in the expression of a specific gene in the specific samples compared to the reference gene *actin*. The visualized bands corresponding to the HSP90 protein were identified as described in Section 3. In total, 15 μg of proteins were loaded onto the gel. MW—molecular weight standard (Thermo Scientific PageRuler Prestained Protein Ladder). A.U.—arbitrary units. The statistical differences between the cv. Delisa and its mutant 522DK (Student’s *t*-test, *p* ≤ 0.05) and between the cv. Bowman and its mutants (Duncan’s test, *p* ≤ 0.05) for each temperature are indicated by different letters. Additionally, the accumulation of the transcript and protein in the Delisa and Bowman cultivars at different temperatures was also compared. The comparisons were performed in pairs (for 20 °C and 5 °C; 20 °C and 27 °C) (Student’s *t*-test, *p* ≤ 0.05), and the statistical differences are indicated by an asterisk.

In cells, heat-shock proteins can be found in the cytoplasm, in specific organelles such as the mitochondria or the endoplasmic reticulum, and, generally, in the cell membranes. The localization can be different for specific HSPs. Our studies on barley enabled us to prove the presence of HSP90 in a membrane fraction that was isolated from barley plants (Figure 1C–F); however, it was below the detection limit in the cytosolic fraction for our method. According to Xu et al. [21], HSP90 mainly occurs in the cytoplasm and only sometimes in the endoplasmic reticulum, mitochondria and chloroplasts, but recent studies showed that HSP90 can also interact with the cell membranes and affect their structure [22]. 

**Figure 2:**
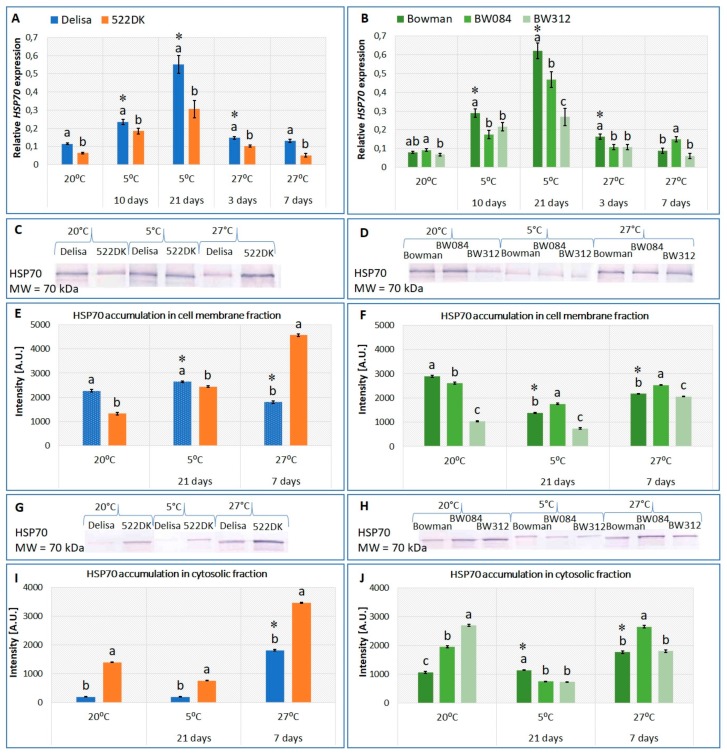
Changes in the relative transcript level of *HSP70* (**A**,**B**) and the accumulation of the HSP70 protein in the barley cell membrane fraction (**C**–**F**) and in the cytosolic fraction (**G**–**J**) of the cv. Delisa, 522DK mutant, cv. Bowman, and the BW084 and BW312 mutants growing in different temperature conditions. The transcript levels are presented as the fold change in the expression of a specific gene in the specific samples compared to the reference gene *actin*. The visualized bands correspond to the HSP70 protein identified as described in Section 3. In total, 10 μg of the proteins from the cell membrane fraction and the cytosolic fractions were loaded onto the gel. MW—molecular weight standard (Thermo Scientific PageRuler Prestained Protein Ladder). A.U.—arbitrary units. Statistical differences between the cv. Delisa and its mutant 522DK (Student’s *t*-test, *p* ≤ 0.05) and between the cv. Bowman and its mutants (Duncan’s test, *p* ≤ 0.05) for each temperature are indicated by different letters. Additionally, the accumulations of the transcript and protein in the Delisa and Bowman cultivars at different temperatures were also compared. The comparisons were performed in pairs (for 20 °C and 5 °C; 20 °C and 27 °C) (Student’s *t*-test, *p* ≤ 0.05) and the statistical differences are indicated by an asterisk.

On the other hand, immunodetecting the HSP70 protein using the primary antibody against cytoplasmic HSP70 revealed the presence of this protein in both the membrane and the cytosolic fractions that were isolated from barley (Figure 2C–J). This finding is in agreement with the literature. HSP70 was originally identified as being one of the most abundant proteins produced in response to an elevated temperature that induces thermotolerance [23]. Currently, these 70-kDa heat-shock proteins are known to be upregulated during many cellular stresses, and they are essential during normal growth. HSP70 is a multigenic family, and the various members that constitute this family are present in different cellular compartments [9,24]. Recently, it was found that the HSP70 protein incorporates into the lipid bilayer and interacts with the cell membranes [25]. It was explained that the insertion of HSP70 into the plasma membrane is the gateway for its export to the extracellular matrix in the form of vesicles, where they play a signaling role to alert and activate the necessary defense machinery against harmful conditions.

As for the sHSPs, in our study, HSP17 and HSP18 were not found in the cell membrane or cytosolic fraction of the barley, although the *sHSP* transcripts were present and even drastically increased after acclimation at 27 °C. It is likely that these proteins typically accumulate under extreme high-temperature stress, usually higher than 40 °C [26,27,28]. The high accumulation of the *sHSP* transcripts in plants might be interpreted as a mechanism that prepares plants for more extreme temperature. Simultaneously, as discussed by Dhaubhadel et al. [29], the presence of a specific messenger RNA (mRNA) of HSPs in cells is not necessarily accompanied by an active translation or a translation that is in accordance with their levels. According to the literature, sHSPs are located rather in the thylakoid membranes where they can act as “membrane-stabilizing factors” by influencing the integrity of the cell membrane during stress [6,10,12]. 

### 2.2. Changes in the Accumulation of the HSP90 Transcript and Protein in the Barley BR Mutants and Wild-Type Plants Growing at 20 °C and Acclimated at 5 °C and 27 °C

In our experiment, the acclimation of the cultivar Delisa at 5 °C did not cause any significant differences in the accumulation of the *HSP90* transcript compared to the plants in the control conditions (20 °C) (Figure 1A). The barley cultivar Bowman at 5 °C was characterized by an increased accumulation of the *HSP90* transcript compared to the plants at 20 °C (Figure 1B), which is in agreement with the findings of Krishna et al. [30] and Kagale et al. [31]. These authors observed an increase in the accumulation of the *HSP90* transcript in *Brassica napus* seedlings at 5 °C and in *Arabidopsis thaliana* at 2 °C. 

In our barley, the accumulation of the HSP90 protein in the cell membranes was significantly lower after 21 days at 5 °C than in the respective plants at 20 °C in both the Delisa and the Bowman cultivars (Figure 1C–F). Earlier, Krishna et al. [30] observed a gradual increase in the accumulation of the HSP90 protein in the leaf tissue of *B. napus* at 5 °C, while Vítámvás et al. [32] found that, in winter wheat, the accumulation of the HSP90 protein decreased at 6 °C if compared to the control plants.

As for studies of BR mutants, a decrease in the accumulation of the *HSP* transcript in the BR mutants (compared to the wild types) was sometimes accompanied by an increase in the accumulation of the protein but sometimes not (Figure 1 A–F). For example, the level of the *HSP90* transcript was lower in the 522DK than in the Delisa at 20 °C and 5 °C (after 21 days), while the protein accumulation in the membrane fraction was doubled in this mutant in comparison to wild type. Simultaneously, at 5 °C and 20 °C, a lower accumulation of the *HSP90* transcript in BW312 (compared to the Bowman) was also accompanied by a lower accumulation of the protein. Hence, an interesting phenomenon can be observed; the disturbances in BR biosynthesis (in the BW084 and 522DK mutants) were reflected by a decreased accumulation of the *HSP90* transcript after a shorter or longer exposure to the low temperature, respectively (effect at the transcriptional level) (Figure 1A,B). Interestingly, the disturbance in BR biosynthesis in the 522DK mutant resulted in an increase in the HSP90 protein accumulation under both control (20 °C) and stress conditions (5 °C) for the Delisa cultivar (effect at the translational level) (Figure 1C,E). However, the mutation in the BW084 mutant did not have any significant effect at 20 °C (or had only a weak effect at 5 °C) at the translational level (Figure 1D,F). On the other hand, a disturbance in the BR signaling in the BW312 mutant led to a significant decrease in the transcript and protein accumulations compared to the Bowman cultivar under at control and low temperature (Figure 1B,D,F). Thus, the disturbance in BR signaling had an effect at both transcriptional and translational levels. It can also be inferred that the BR insensitivity affects the function of the major transcription factors that are required for the BR-dependent gene [33,34,35]. To the best of our knowledge, there is only one article that discussed the effect of BR on the expression of HSP90 in plants at low temperature. *Arabidopsis thaliana* that was acclimated at 2 °C (three days) and treated with 1 μM 24-epibrassinolide had a similar accumulation of the *HSP90* transcript level to the BR-untreated plants [31].

Furthermore, in our experiment, the *HSP90* transcript and the HSP90 protein levels were analyzed in both cultivars that were acclimated at a temperature of 27 °C. In the Bowman and Delisa, after a transient increase in the accumulation of the *HSP90* transcript on the third day of the acclimation at 27 °C (statistically significant effect only in the Bowman), there was a lower level of this transcript on the seventh day (Figure 1A,B). On the seventh day of the acclimation, the HSP90 protein accumulation in the cell membranes was about 30% lower in the Delisa but was higher in the Bowman compared to the control (20 °C) (Figure 1C–F). From the literature, it is well known that high temperatures stimulate the synthesis of HSPs in plants [5]. The known effects are primarily related to the effects of extremely high temperatures (heat shock). The expression of *HSP90* genes was higher in carrot cell lines at a temperature of 38 °C [36]. Pavli et al. [37] investigated the effect of heat stress (47 °C; 180 min) on the expression of *HSP90* in four sorghum genotypes and found that an increase in the relative accumulation of the *HSP90* transcript was followed by a decrease. In the leaves of *Brassica napus*, heat (45 °C; 2 h) increased the accumulation of the *HSP90* transcript, as well as the accumulation of the HSP90 protein [30]. In this context our studies provide some new knowledge about the expression of HSP90 during the process of acclimation at slightly lower temperatures, which may lead to an acquired thermotolerance as mentioned by Altschuler and Mascarenhas [3]. 

Furthermore, we observed how disturbances in BR signaling or BR deficiency modified the patterns of the changes that were described for the wild-type cultivars at an acclimating temperature of 27 °C. In the 522DK mutant, there was no impact of the BR deficiency on the accumulation of *HSP90*, especially after three days of acclimation at 27 °C. After seven days of acclimation, 522DK accumulated about 15% more of the *HSP90* transcript than its wild-type cultivar (Figure 1A). No difference in the protein content was observed between the wild type and the mutant (Figure 1C,E). After both three and seven days of acclimation at 27 °C, BW084 accumulated about 35% and 28% less of the *HSP90* transcript, respectively, than the wild-type Bowman, while the accumulation of the HSP90 protein was about 60% higher than in the Bowman. For BW312, the accumulation of the HSP90 protein (similar to the transcript; Figure 1B) was about 20% lower than in the Bowman after seven days at 27 °C (Figure 1D,F). 

Although BRs are known to play a role in enhancing the high-temperature stress tolerance in plants [38], the available data regarding their influence on the expression of HSP90 are different. Dhaubhadel et al. [29,39] reported that, after heat stress (*Brassica napus* and tomato), the accumulations of HSPs (including HSP90) at the transcript and protein levels were higher in the BR-treated plants than in the untreated control. Hovewer, Kagale et al. [31] later reported that the exogenous application of BRs in wild-type *Arabidopsis* exposed to a high temperature did not change the accumulation of the *HSP90* transcript compared to the BR-untreated plants. Since there is no information on the effect of BR on the expression of HSP90 during the process of acclimation at slightly lower temperatures, our studies provide new knowledge on this subject. 

### 2.3. Changes in the Accumulation of the HSP70 Transcript and Protein in the Barley BR Mutants and Wild-Type Plants Growing at 20 °C and Acclimated at 5 °C and 27 °C

Both the Delisa and the Bowman cultivars accumulated significantly more of the *HSP70* transcript after 10/21 days of acclimation at 5 °C than at 20 °C (Figure 2A,B). After acclimation at 5 °C, the Delisa accumulated about 13% more of the HSP70 protein in the cell membrane fraction than the control plants (20 °C) (Figure 2C,E). In contrast to the Delisa, the cold-acclimated Bowman accumulated about 50% less of the HSP70 protein in the cell membrane fraction than the plants that were not acclimated (Figure 2D,F). In the cytosolic fraction, the HSP70 accumulation did not differ for the Delisa growing at 5 °C and 20 °C (Figure 2G,I). The HSP70 accumulation was statistically significantly higher for the cold-acclimated Bowman compared to the plants growing at 20 °C, although only by about 9% (Figure 2H,J). The results of our study correspond to the effects that were described in the literature, especially in the case of the accumulation of the *HSP70* transcript. The effect of temperature on the transcript and protein levels of HSP70 in three tomato genotypes was investigated by Kubienová et al. [23]. A temperature of 4 °C increased the accumulation of the *HSP70* transcript in the two genotypes and also increased the accumulation of the HSP70 protein in one of the three genotypes. The cold acclimation (6 °C) of winter wheat (*Triticum aestivum* L.) increased the accumulation of the HSP70 protein in a crude extract of crowns [32]. Similar results were also obtained on rice roots that were cold acclimated at 10 °C [40], pea stems (10/2 °C; day/night) [41], and chicory roots [42]. 

To the best of our knowledge, there were no studies concerning the effect of BR on the accumulation of HSP70 in cold-acclimated plants. The results of our study show that both BR-deficient mutants, 522DK and BW084, accumulated significantly less of the *HSP70* transcript (by about 44% and 24%, respectively) after 21 days of cold acclimation than their respective wild types (Figure 2A,B). The BR-signaling mutant BW312 was also characterized by a markedly lower level of the accumulation of the *HSP70* transcript after both 10 and 21 days of cold compared to the Bowman cultivar (Figure 2B). The accumulation of the HSP70 protein in the cell membrane fraction was lower in the 522DK mutant than in the Delisa both at 20 °C and after acclimation at 5 °C (Figure 2C,E). However, the 522DK mutant accumulated significantly more of the HSP70 protein in the cytosolic fraction than its wild type at 20 °C and 5 °C (Figure 2G,I). The accumulation of the HSP70 protein in the cell membrane fraction of BW084 was lower than in the Bowman at 20 °C but higher than in the Bowman after growing at 5 °C (Figure 2D,F). The opposite tendency was observed in the cytosolic fraction in which HSP70 was accumulated in a higher amount in BW084 than in the Bowman at 20 °C but in a lower amount in BW084 than in the Bowman at 5 °C (Figure 2H,J). Compared to the Bowman (at 20 °C), the BW312 mutant was characterized by about a 70% lower accumulation of HSP70 in the cell membrane fraction, while there was an increase of about 70% in the accumulation of this protein in the cytosolic fraction (Figure 2D,F,H,J). After acclimation at 5 °C, the accumulation of HSP70 in this mutant was lower compared to the wild type in both the membrane and the cytosolic fraction (Figure 2D,F,H,J). 

In both the Delisa and the Bowman cultivars, three days of acclimation at 27 °C increased the accumulation of the *HSP70* transcript compared to the control (20 °C); however, after seven days of acclimation, its level returned to that of the control plants (Figure 2A,B). In the case of the accumulation of the HSP70 protein, a different tendency was observed for the cell membrane and cytosolic fractions of the Delisa and Bowman. In the cell membrane fraction, the HSP70 levels were significantly lower than in the control plants (20 °C) (Figure 2C–F). In the cytosolic fraction, there was a noticeable increase in both the Delisa and the Bowman cultivars (Figure 2G–J). As mentioned earlier, high temperatures usually increase the accumulation of the *HSP70* transcript and protein in plants, but this is mainly associated with more extreme temperatures than those tested in our studies. Heat stress (40.5 °C) increased the accumulation of the HSP70 transcript and protein in tomato compared to the control (20 °C) [23]. After one hour at 37 °C, there was a significant increase for all 10 studied members in the stress 70 family in spinach and tomato seedlings, which was followed by a decrease during the next 11 h of heat shock treatment [43]. 

In our work, we also investigated the effect of a temperature of 27 °C on the expression of HSP70 in the BR mutants. The BR-deficient mutant 522DK that was acclimated at 27 °C for three and seven days accumulated significantly less of the *HSP70* transcript than its wild-type Delisa (Figure 2A). A similar tendency was observed for the BW084 mutant that was acclimated to a high temperature for three days; however, after seven days of growing at 27 °C, the *HSP70* transcript level was higher in this mutant than in the wild-type Bowman (Figure 2B). Despite some differences in the accumulation of the transcript in both of the BR-deficient mutants at 27 °C, the accumulation of the HSP70 protein was significantly higher in the cell membrane fraction and cytosolic fraction in both mutants compared to their respective wild types (Figure 2C–J). 

After acclimation at 27 °C, the BR-signaling mutant (BW312) had generally a decreased accumulation of the *HSP70* transcript compared to the wild type (Figure 2B). The mutant also accumulated less of the HSP70 protein in the cell membrane fraction than the Bowman, while the HSP70 level in the cytosolic fraction was similar to that in the wild type (Figure 2D,F,H,J).

The significance of BR in regulating the expression of HSP70 under heat stress was also studied by Dhaubhadel et al. [29] and Kagale et al. [31], but for temperatures much higher than 27 °C. The BR-deficient mutants of *Arabidopsis thaliana* at 43 °C accumulated more of the *HSP70* transcript than the unstressed plants, while, in the mutants that were treated with 24-epibrassinolide, the accumulation of the *HSP70* transcript was lower compared to the BR-untreated plants. As for the accumulation of the HSP70 protein, Dhaubhadel et al. [29] showed that treating *Brassica napus* plants with BR under heat stress (45 °C) increased the accumulation of HSP70 compared to the untreated plants. 

Regarding HSP70, we also observed a changed relationship between the presence of HSP70 in the cytosolic fraction and membrane fraction for both the mutants and the wild types. Earlier, Armijo et al. [25] also found that HSP70 can be incorporated into the lipid bilayer in artificial cell membranes. In the mutants that were cultured especially at 20 °C, the relationship between HSP70 that was present in the cytosolic fraction and HSP70 in the membranes was disturbed compared to the wild types (Supplementary Figure S1,). All of the mutants accumulated more HSP70 in the cytosolic fraction than in the membrane fraction at 20 °C compared to the wild types. This relationship was also slightly changed at 5 °C, although it was unchanged at 27 °C (data not shown). This may suggest that BRs somehow participate in regulating the balance between the HSP70 present in the cytoplasm and that incorporated into the membranes; however, once again, because the phenomenon is dependent on temperature, it requires more detailed studies. 

### 2.4. Changes in the Accumulation of the HSP18 and HSP17 Transcripts in the Barley BR Mutants and Wild-Type Plants Growing at 20 °C and Acclimated at 5 °C and 27 °C

**Figure 3:**
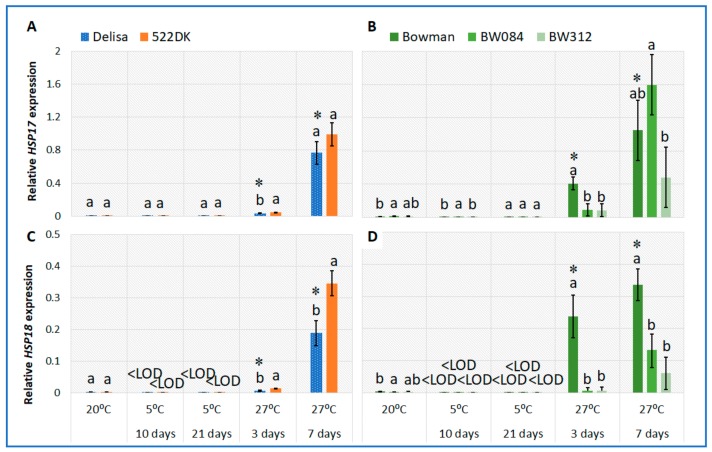
Relative transcript level of *HSP17* and *HSP18* in the barley leaves of the cv. Delisa, 522DK mutant (**A**,**C**), cv. Bowman, and the BW084 and BW312 (**B**,**D**) mutants at 20 °C and during and after acclimation at 5 °C and 27 °C. The transcript levels are presented as the fold change in the expression of a specific gene in the specific samples compared to the reference gene *actin*. The statistical differences between the cv. Delisa and its mutant 522DK (Student’s *t*-test, *p* ≤ 0.05) and between cv. Bowman and its mutants (Duncan’s test, *p* ≤ 0.05) for each temperature are indicated by different letters. Additionally, the accumulations of the transcript in the Delisa and Bowman cultivars at different temperatures were compared. The comparisons were performed in pairs (for 20 °C and 5 °C; 20 °C and 27 °C) (Student’s *t*-test, *p* ≤ 0.05) and the statistical differences are indicated by an asterisk. LOD: below detection limit.

In our studies, the acclimation of the barley cv. Delisa and Bowman at a low temperature did not change the accumulation of the *HSP17* transcript compared to the control growing at 20 °C (Figure 3A,B), and only trace amounts of the transcript were detected at both temperatures. Transcript *HSP18* was below the detection limit in plants at 5 °C, although some traces were observed at 20 °C (control) (Figure 3C,D). No accumulation of the HSP17 and HSP18 proteins was detected in either the cell membranes or in the cytosolic fraction of the plants that were acclimated to a low temperature. According to the literature, however, the connection between cold treatment and the expression of sHSPs in some plant species may exist. For example, sHSPs take part in the mechanisms of tomato chilling tolerance [44]. Moreover, the overexpression of the chloroplast-localized sHSPs increased the cold tolerance in tomato [45]. On the other hand, the role of BRs in the expression of the small heat shock protein during a plant’s acclimation to a low temperature is unknown. In light of this, we assume that our studies are the first to present the slight differences in the level of accumulated transcripts that were only found between the Bowman and its mutants (not between the Delisa and 522DK) (Figure 3A–D). In the control conditions of 20 °C, the accumulation of the *HSP17* and *HSP18* transcripts was slightly lower in the Bowman than in its mutants. At a low temperature, only the *HSP17* transcript was observed, and only during acclimation (10th day) was a slightly increased amount observed in the case of BW084 (compared to the Bowman). This effect disappeared after 21 days in cold. 

Heat stress intensively induces the accumulation of the sHSP transcript and protein in plants [5,27,28,46,47,48]. The temperatures that were tested were within a range of 37–45 °C, but less is known about the expression of the sHSP during the process of plant acclimation in which a gradual increase in the temperature improves the thermotolerance of plants to extremely high temperatures [4]. In our studies, during acclimation at 27 °C, the Delisa and Bowman accumulated significantly more of the *HSP18* and *HSP17* transcripts compared to the plants in the control conditions (20 °C) (Figure 3A–D). Interestingly, the effect was really strong after seven days of acclimation and much weaker after three days, especially in the Delisa. The plant reaction was then much slower than in the case of heat shock (described in literature), where there was an increase in the transcript accumulation after several hours. Moreover, it is likely that the plants at 27 °C had an increased transcript accumulation of the sHSP as part of the preliminary acclimation mechanisms that prepare plants for more extreme temperatures. As was explained at the beginning of this section, we were not able to detect any of the small heat-shock proteins in the isolated cytosolic or membrane fractions.

In our studies, we also focused on changes in *sHSP* during the acclimation of the BR mutants at 27 °C. After three days, the transcript level (*HSP17* and *HSP18*) in the BR-deficient mutant 522DK was higher than in the wild-type Delisa (Figure 3A,C). After seven days, this tendency was maintained, although only in the case of *HSP18* was the difference between 522DK and the Delisa statistically significant. In contrast to 522DK, after three days of acclimation, the second BR-deficient mutant had a significantly lower accumulation of the *HSP17* and *HSP18* transcripts compared to its wild type (Figure 3B,D). After seven days, the effect was only maintained in the case of *HSP18*. The BR-signaling mutant BW312 had a drastically lower accumulation of both transcripts compared to the wild type after three and seven days of acclimation (Figure 3B,D). As already mentioned for the mutants, we also did not detect any HSP17 and HSP18 proteins in the cytosolic or membrane fractions. Although we realize that it can be difficult to visualize these proteins due to their low abundance and a significant increase in their content only at high temperatures, we decided to perform such an experiment in order to determine whether a mutation in the BR synthesis or BR signaling in barley plants can affect the expression of the studied sHSPs. 

According to the literature, BRs are thought to be a factor that induces sHSP synthesis in heat-stressed plants [31,39,49], but these studies were performed only for extremely high temperatures. The 24-epibrassinolide-treated *Brassica napus* seedlings accumulated more *sHSP* transcripts at 45 °C than the untreated ones [39]. BR-treated tomato accumulated a higher amount of mitochondrial small heat-shock proteins than the BR-untreated control at 38 °C [49]. Kagale et al. [31] studied the effect of 43 °C on the accumulation of the class II *sHSP* transcript in two BR-deficient mutants of *Arabidopsis thaliana.* One of the mutants accumulated *sHSP* even before heat stress (in the control conditions of 22 °C), while the second one did not. A high temperature increased the accumulation of the *sHSP* transcript in both mutants compared to the plants in the control conditions (22 °C). Interestingly, treating the mutants with 24-epibrassinolide even decreased the accumulation of the *sHSP* transcript in both mutants. 

### 2.5. General Comments

The role of brassinosteroids as positive regulators of the expression of HSP in barley seems to be proven mainly by the results that were obtained for the BR-signaling mutant. In most cases, the mutant with a defective BR receptor had a lower accumulation of the *HSP* transcripts and HSP proteins compared to the wild type regardless of the plant growth/acclimation temperature. The results that were obtained for both BR-deficient mutants (BW084 and 522DK) confirm that BRs are among the players that regulate HSP expression. Interestingly, however, the accumulation of the *HSP70* or *HSP90* transcripts was most often lower in the mutants, although this was also often accompanied by an increased level of the heat-shock proteins. According to the literature [29], the levels of heat-shock protein may be higher in heat-treated plants (during the recovery period), while the transcripts that correspond to these proteins can be present at higher levels in an untreated control. In addition, our results shows that, contrary to the disturbances in BR signaling, a decrease in the level of BRs (BRs were drastically lowered in BW084, while relatively slightly lowered in 522DK) did not always act negatively on the accumulation of the HSP protein. What is more surprising, however, is that there were some cases in which the direction of the changes was different in 522DK and BW084. For example, the level of HSP90 at 5 °C was higher in the 522DK mutant than in the wild type (Delisa), while, in BW084, it was slightly lower than in its wild type (Figure 1C–F). The differences in the transcript and protein accumulation patterns that were observed between both BR biosynthetic mutants seem to reflect the fact that the mutants have defects at different stages of the BR biosynthesis pathway [19]. The 522DK mutant carried the missense mutations in the *HvDWARF* gene, while, in BW084, it was in the *HvCPD* gene. *HvDWARF* encodes the C6-oxidase enzyme that is involved in the later stages of BR biosynthesis, while the enzyme that is encoded by *HvCPD* acts during the earlier BR biosynthetic stages. The phenomenon of the different expressions of HSP depending on the mutation that was connected to BR biosynthesis was also observed by Kagale et al. [31]. At 22 °C, the *Arabidopsis* BR-deficient mutant *det2-1* accumulated *HSP* transcripts, while a second BR-deficient mutant *dwf4* did not. Further, the influence of the genetic background cannot be underestimated, since both mutants come from different cultivars, which are characterized by different levels of other hormones [17]. Because BRs, when regulating the physiological/biochemical processes, act in a complicated network with other hormones (and also interact with them), the regulatory mechanism of biosynthesis of some proteins including HSP may be more complex. 

The directions of changes of the accumulation of the transcripts and proteins of the HSP group in mutants compared to the wild types at various temperatures are summarized and visualized in Supplementary Tables S1 and S2. 

The plant growth temperature (20 °C, control) as well as the acclimation of plants at 5 °C or 27 °C, affected the expression of the HSP in all of the mutants. Changes in the expression of the selected heat-shock proteins during the acclimation of mutants may, however, only partly explain the reasons for the altered tolerance of the mutants to a high temperature or frost that was described in our earlier studies [17]. In relation to the tolerance to frost, attention should be paid, in particular, to changes in the expression of HSP90 observed during acclimation. After acclimation at 5 °C, the BW084 and BW312 mutants were characterized by a lower survival rate in frost (−6 °C and −8 °C) than the Bowman reference cultivar [17]. At the same time, these mutants accumulated less of the protective HSP90 protein during acclimation at 5 °C. HSP90 is indicated as being important for low-temperature tolerance [30]. The 522DK mutant accumulated more HSP90 after acclimation than the Delisa and had an unchanged (at −8 °C) or even a slightly higher frost tolerance (−6 °C) than the wild type [17]. It is worth mentioning that, compared to BW312 or BW084, 522DK also accumulated more HSP90 before acclimation at 5 °C (Figure 1C–F). This could also have some protective significance for the plants at the beginning of cold acclimation.

In turn, in the case of the higher tolerance of the mutants to a high temperature (38–45 °C) than the wild type [17], more attention should be paid to changes in the HSP70 protein level, particularly in the mutants of BR biosynthesis. Both of these mutants accumulated more HSP70 than the wild type after acclimation at 27 °C. This tendency, however, did not apply to the BR-signaling mutant, in which the HSP70 level was similar to the wild-type Bowman, although the tolerance to a high temperature was higher, thus other factors had to be additionally responsible for its higher tolerance. 

The main findings of the whole work are summarized/concluded in Section 4. 

## 3. Materials and Methods 

### 3.1. Plant Material

The spring barley (*Hordeum vulgare* L.) cultivars (Delisa and Bowman) and their mutants (522DK, BW084, and BW312) were used in our study. The mutants were derived from the collection of the Department of Genetics at the University of Silesia (Katowice, Poland). Both the cultivars and the mutants were selected according to the works by Gruszka et al. [18] and Dockter et al. [19]. 

### 3.2. Plant Culture and Experimental Design

The plants were cultivated as described by Sadura et al. [17]. Briefly, after germination, the plants were cultivated in a growth chamber at 20 °C (16 h photoperiod) for about three weeks and then the plants were divided into two groups. The first group was acclimated at 5 °C (day/night) under an 8-h photoperiod for 21 days, while the second group was acclimated at 27 °C (day/night) under a 16-h photoperiod for seven days. After acclimation, the plants had four well-developed leaves and sometimes a young fifth leaf. Light intensity in the growth chambers was 170 μmol·m^−1^·s^−1^ (HPS Philips SON-T AGRO 400-W lamps). 

Samples for the analyses of the accumulation of a transcript (*HSP90*, *HSP70*, *HSP18*, and *HSP17*) were taken from the plants (the middle part of the second leaf) at 20 °C (control, before acclimation) and during/after acclimation (on the 10th and 21st days at 5 °C; on the third and seventh days at 27 °C). 

To determine the accumulation of protein (HSP90, HSP70, HSP18, and HSP17), two types of samples were prepared: cytosolic fraction samples and membrane fraction samples. The aerial parts of the seedlings were cut off and the cell membrane fraction and cytosolic fraction were immediately isolated and then frozen at −80 °C for further analysis of the proteins. The samples were taken at 20 °C (control, before acclimation) and after acclimation (on the 21st day at 5 °C and on the seventh day at 27 °C).

### 3.3. Isolation of the Membrane and Cytosolic Fractions

The cell membranes were isolated according to a modified protocol of Sommarin et al. [50] and Janeczko et al. [51]. In total, 100 g of leaves were homogenized in 400 mL of a cell fraction isolation buffer (pH 7.8) containing 10 mM Tris/HCl, 0.25 M sucrose, 1 mM ethylene diaminetetraacetic acid (EDTA), and 2.5 mM dithiotreitol (DTT) (Sigma-Aldrich, Poznań, Poland) using a Camry CR 4050 blender. The crude extract was filtered through two layers of fiber and centrifuged for 10 min at 10,000× *g* (Beckman L3-50, Beckman Coulter, Palo Alto, CA, USA) in order to eliminate any residues after plant extraction. The supernatant was centrifuged for 30 min at 80,000× *g* (Beckman L8-M, Beckman Coulter, Palo Alto, CA, USA). The obtained pellet was the cell membrane fraction, and the supernatant was considered to be the cytosolic fraction. Then, 15 mL of the supernatant was densified with 2.5 g of Sephadex for 30 min. The samples that were obtained were used to analyze the accumulation of protein.

### 3.4. Accumulation of the Transcripts of HSP90, HSP70, HSP18, and HSP17: RNA Isolation, Complementary DNA (cDNA) Synthesis, and Real-Time PCR Reaction

The accumulation of the transcripts of *HSP90*, *HSP70*, *HSP18*, and *HSP17* was determined using quantitative PCR amplification and analyzed using a 7500 Real-Time PCR System (Applied Biosystems, Foster City, CA, USA). The collected samples (approximately 0.05 g of the central part of the second leaf) were frozen in liquid nitrogen and then the mRNA was isolated using an RNeasy Plant Mini Kit (Qiagen, Hilden, Germany). The concentration and quality of the mRNA was assessed using a Q5000 UV–Vis Spectrophotometer (Quawell, San Jose, CA, USA). Next, the genomic DNA was eliminated by adding 2 μL of mRNA (approximately 600 ng) to a mixture of 2 μL of a genomic DNA (gDNA) Wipeout Buffer and 10 μL of RNase-free water (included in the QuantiTectReverse Transcription (RT) Kit, Qiagen, Hilden, Germany). After a 2-min incubation at 42 °C, the template RNA that was obtained was added to a reverse-transcription master mix (containing 1 μL of Quantiscript Reverse Transcriptase, 4 μL of a Quantiscript RT Buffer, and 1 μL of RT Primer Mix (QuantiTectReverse Transcription Kit, Qiagen, Hilden, Germany), and the reverse-transcription reaction was performed. The concentration and quality of the RNA and cDNA were assessed using a Q5000 UV–Vis spectrophotometer (Quawell, San Jose, CA, United States). The PCR amplifications of the *HSP90*, *HSP70*, *HSP18*, and *HSP17* transcripts were run in triplicate as described by Jurczyk et al. [52]. The primers and probes were designed using Primer Express Software v 3.0.1 (Applied Biosystems by Life Technologies, Foster City, CA, USA). The primer and probe sequences are listed in Table 1. The levels of the *HSP90*, *HSP70*, *HSP18*, and *HSP17* transcripts were determined relative to *actin* as the reference gene [53]. Five biological replicates (analyses for different cDNAs) were made.

### 3.5. Analysis of the Protein Content in the Cell Membrane and the Cytosolic Fractions

The protein content was determined based on the Sedmak and Grossberg [54] procedure. Firstly, 2 μL of the microsomal fraction was mixed with 2 μL of a 10% water solution of Triton X-100 (Sigma-Aldrich, Poznań, Poland) and 196 μL of the cell fraction isolation buffer before being kept on ice for 15 min. Then, 3 mL of water and 1 mL of Bradford reagent (BioRad, Munich, Germany) were added. After 10 min, the absorbance was measured (596 nm) using a UV–Vis spectrometer Lambda Bio 20 (Perkin Elmer, Waltham, MA, USA). The measurements were performed in triplicate. Bovine serum albumin (BSA) (Sigma-Aldrich, Poznań, Poland) was used as the calibration standard. The BSA was diluted in the cell fraction isolation buffer and 2 μL of a 10% water solution of Triton X-100 was added. For the cytosolic fraction, 185 μL of the cell fraction isolation buffer, 15 μL of the cytosolic fraction, 3 mL of water, and 1 mL of Bradford reagent were mixed. The absorbance was then measured (596 nm).

### 3.6. Analysis of the Accumulation of HSP90, HSP70, HSP18.5, and HSP17.7 Using Immunoblotting

The same amount of proteins (selected after optimization and testing sample dilutions in range of 2.5 - 30 μg), which were isolated from the analyzed samples, was loaded and separated on 12% SDS-PAGE (1-mm polyacrylamide gel) based on the Laemmli [55] procedure and blotted to the nitrocellulose membrane (1 h at 45.5 mA (7–9 V)) using a BioRad semi-dry transfer) (Bio-Rad Laboratories, Inc., Hercules, CA, USA). The membranes were blocked with low-fat milk powder that was diluted in a Tris-buffered saline/Tween (TBS-T) buffer (containing 0.9% NaCl and 10 mM Tris) overnight. Subsequently, the membranes were washed four times for 5 min with TBS-T buffer and probed in the appropriate antibodies for 1.5 h (Agrisera, Vännäs, Sweden) (anti-HSP90, 1:3000; anti-HSP70, 1:3000; anti-HSP18.5, 1:1000; anti-HSP17.7, 1:1000). Next, the membranes were washed with a TBS-T buffer (four times for 5 min) and probed in alkaline phosphatase-conjugated secondary anti-rabbit antibody for 1.5 h (Sigma-Aldrich, Poznań, Poland) (dilution: HSP90 1:2000, HSP70 1:3000, HSP18.5 1:4000, HSP17.7 1:2000). Dilutions of all of the antibodies were applied according to the protocol provided by Agrisera after optimization. Three independent trials were performed. The protein content was quantified by a densitometric analysis of the visualized band intensity staining using ImageJ software (NIH, Bethesda, MD, USA). The averages are expressed as arbitrary units (A.U.) correlated with the area under densitometric curves. The densitometry was performed using ImageJ software (NIH, Bethesda, MD, USA). 

### 3.7. Statistical Analysis

The statistical analysis (ANOVA, post hoc test) was done using Statistica 13.1 (StatSoft, Tulsa, OK, USA). Duncan’s test was used to compare more than two groups (Bowman cultivar and its mutants BW084 and BW312). Student’s *t*-test was used to compare two groups (Delisa cultivar and its mutant 522DK). The values marked with the same letters in specific figures did not differ significantly. Additionally, the accumulations of the transcripts and proteins in the Delisa and Bowman cultivars at different temperatures were compared. The comparisons were performed in pairs (for 20 °C and 5 °C; 20 °C and 27 °C) (Student’s *t*-test, *p* ≤ 0.05). The statistical significances are indicated by an asterisk (*). The averages on the figures are presented together with the standard error bars. 

## 4. Conclusions

This work provides information on changes in HSPs in barley wild types and brassinosteroid mutants during plant growth at 20 °C and acclimation at 5 °C or at 27 °C, which usually enables plants to develop a better tolerance to more extreme temperatures. 

The main findings of the work are as follows:

(1) In the tested Delisa and Bowman cultivars, the temperature of the growth/acclimation affected the expression of the HSPs. Acclimation at 5 °C increased the *HSP90* transcript only in the Bowman (compared to 20 °C). Acclimation at 27 °C decreased the *HSP90* transcript and drastically increased the *sHSP* transcripts in both cultivars. Acclimation at 5 °C and 27 °C increased the *HSP70* transcript in both cultivars. As for the respective protein accumulation, the results were more cultivar-dependent, but for both cultivars, identical directions of changes were observed for the accumulation of HSP90 (lower in the membrane fraction at 5 °C) and HSP70 accumulation (lower at 27 °C in the membrane fraction but increased in the cytosolic fraction).
(2) The role of brassinosteroids as positive regulators of the expression of HSPs seems to be proven by the results that were obtained for the BR-signaling mutant. In most cases, the mutant with a defective BR receptor had a lower accumulation of the *HSP* transcripts and HSP proteins compared to the wild type, regardless of the plant growth/acclimation temperature. The results that were obtained for the BR-deficient mutants (BW084 and 522DK) may additionally confirm that BRs are among the players that regulate the expression of HSPs. The results, however, also show that lowering the level of BRs (BRs were drastically lower in BW084, but only relatively slightly lower in 522DK) does not always act negatively on the expression of HSPs. Moreover, the genetic background of cultivars from which the biosynthetic mutants were derived also seems to be important for HSP expression, because BRs may act in complicated network with other phytohormones.

## Figures and Tables

**Table 1:** Sequence origins and primer and probe sequences used in the study. ID—identifier.

Gene Name	GenBank ID	Forward Primer	Reverse Primer	TaqMan MGB Probe
*HSP17*	Y07844.1	CGACACCTTCCGCTCCAT	CGGCCGTCTCGCTGTT	FAM–TCCCGGCGTTCTCT–MGB
*HSP18*	X64561.1	CGTATTCGAGTCGGAGCCATT	TCACAACTGTATTTAGGCTGCAGAA	FAM–CTCGCACACACATCAA–MGB
*HSP70*	L32165.1	CCTCAATGTGGCTAGGATCATCAAT	CCACCCCTCTTGTCCAAACC	FAM–CTGCTGCTGCTATTGC–MGB
*HSP90*	AY325266.1	GTTCAAGGCTGTCCTGTTTGTTC	GTTGTTGGCCTTCTTCTTGTTGTC	FAM–CCCCTTCGACCTCTTC–MGB
*Actin*	AY145451.1	GCAACTGGGATGACATGGAGAAAAT	GCCACACGGAGCTCATTGTA	FAM–CTGGCATCACACTTTC–MGB

## Supplementary Materials

Supplementary Materials can be found at https://www.mdpi.com/1422-0067/21/5/1889/s1.
