# Supplementary material for: HSP Transcript and Protein Accumulation in Brassinosteroid Barley Mutants Acclimated to Low and High Temperatures
Source: Int J Mol Sci. 2020 Mar 10;21(5):1889. doi: 10.3390/ijms21051889 (PMC7084868; doi:10.3390/ijms21051889)
Supplement: Supplementary file 1 [file ijms-21-01889-s001.zip › Figure S1.pdf]

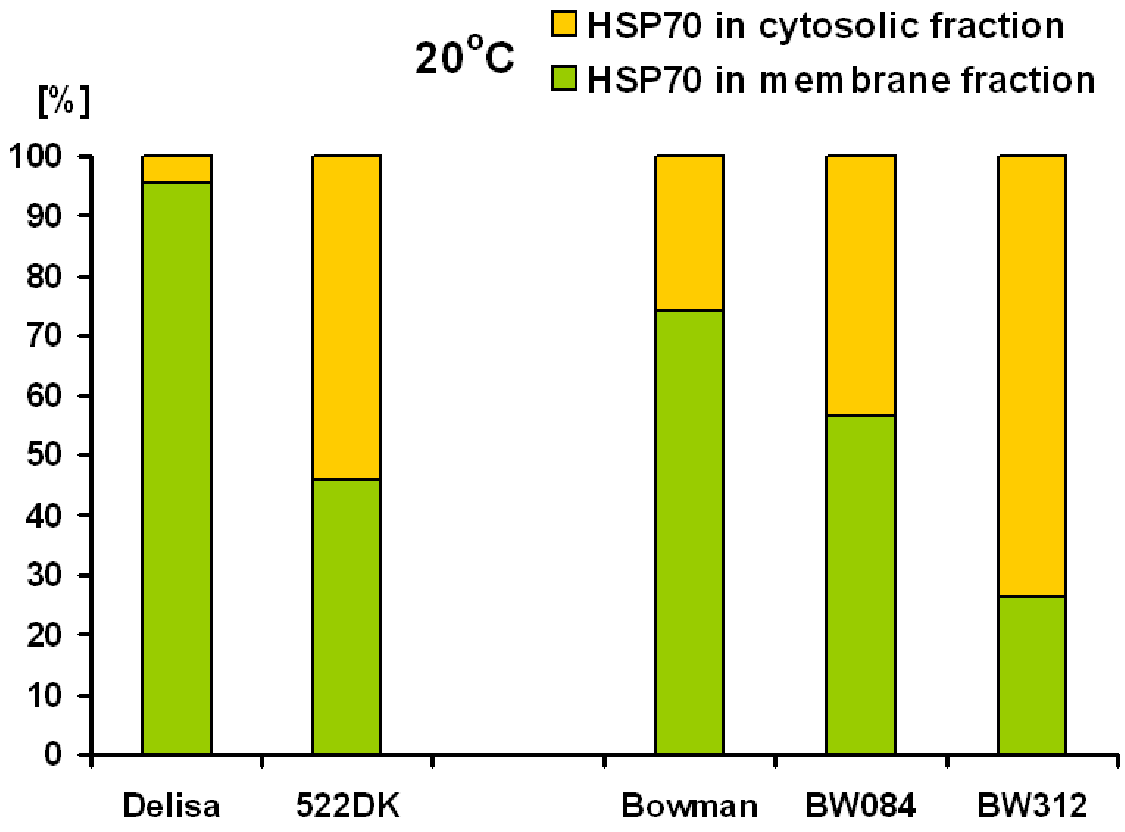

Fig. S1 Percentage relationship of the HSP70 protein in the cytosolic fraction to the HSP70 in the membrane fraction in barley wild types (Delisa and Bowman) and the mutants 522DK, BW084 and BW312 that had been cultured at 20 °C. Data are expressed as the % of the total accumulation of HSP70, which was calculated as the sum of HSP70 in the cytosolic fraction and in the membrane fraction from Figure 2 E, F, I, J.
