# Supplementary material for: HSP Transcript and Protein Accumulation in Brassinosteroid Barley Mutants Acclimated to Low and High Temperatures
Source: Int J Mol Sci. 2020 Mar 10;21(5):1889. doi: 10.3390/ijms21051889 (PMC7084868; doi:10.3390/ijms21051889)
Supplement: Supplementary file 1 [file ijms-21-01889-s001.zip › Table S1.pdf]

Table S1. Changes in the accumulation of the *HSP90, HSP70, HSP18* and *HSP17* transcripts in barley in relation to a mutation and the temperature of growth (+ increase compared to the wild type; – decrease compared to the wild type; NC not changed compared to the wild type). The plant material in our studies included the barley (*Hordeum vulgare* L.) BR-deficient mutant 522DK (mutation *HvDWARF*) from the Delisa cultivar, the barley BR-deficient mutant BW084 (mutation in the *HvCPD* gene**)**, the BR-signalling defective mutant (BW312, mutation in the *HvBRI1* gene) and their reference cultivar Bowman. < LOD below limit of detection

**Transcript**	**Genetic mutation**														
	HvDWARF					***HvCPD***					***HvBRI1***				
	20 ℃	5 ℃ (10 days)	5 ℃ (21 days)	27 ℃ (3 days)	27 ℃ (7 days)	20 ℃	5 ℃ (10 days)	5 ℃ (21 days)	27 ℃ (3 days)	27 ℃ (7 days)	20 ℃	5 ℃ (10 days)	5 ℃ (21 days)	27 ℃ (3 days)	27 ℃ (7 days)
HSP90	**-**	**NC**	**-**	**NC**	**+**	**NC**	**-**	**NC**	**-**	**-**	**-**	**-**	**-**	**-**	**-**
HSP70	**-**	**-**	**-**	**-**	**-**	**NC**	**-**	**-**	**-**	**+**	**NC**	**-**	**-**	**-**	**NC**
HSP18	**NC**	**<LOD**	**<LOD**	**+**	**+**	**+**	**<LOD**	**<LOD**	**-**	**-**	**NC**	**<LOD**	**<LOD**	**-**	**-**
HSP17	**NC**	**NC**	**NC**	**+**	**NC**	**+**	**+**	**NC**	**-**	**NC**	**NC**	**NC**	**NC**	**-**	**NC**
