# Supplementary material for: HSP Transcript and Protein Accumulation in Brassinosteroid Barley Mutants Acclimated to Low and High Temperatures
Source: Int J Mol Sci. 2020 Mar 10;21(5):1889. doi: 10.3390/ijms21051889 (PMC7084868; doi:10.3390/ijms21051889)
Supplement: Supplementary file 1 [file ijms-21-01889-s001.zip › Table S2.pdf]

Table S2. Changes in the accumulation of the HSP90 and HSP70 proteins in barley in relation to a mutation and the temperature of growth (+ increase compared to the wild type; – decrease compared to the wild type; NC not changed compared to the wild type). The plant material in our studies included the barley (*Hordeum vulgare* L.) BR-deficient mutant 522DK (mutation in the *HvDWARF* gene) from the Delisa cultivar, the barley BR-deficient mutant BW084 (mutation in the *HvCPD* gene**)**, the BR-signalling defective mutant (BW312, mutation in the *HvBRI1* gene) and their reference cultivar Bowman.

**Protein**	**Genetic mutation**								
	HvDWARF			***HvCPD***			***HvBRI1***		
	20 °C	5 °C	27 °C	20 °C	5 °C	27 °C	20 °C	5 °C	27 °C
**HSP90** (cell membrane)	+	+	**NC**	**NC**	-	+	-	-	-
**HSP70** (cell membrane)	-	-	+	-	+	+	-	-	-
**HSP70** (cytosolic fraction)	+	+	+	+	-	+	+	-	**NC**
